# Ube2S regulates Wnt/β-catenin signaling and promotes the progression of non-small cell lung cancer

**DOI:** 10.7150/ijms.40243

**Published:** 2020-01-14

**Authors:** Yanan Qin, Jiang Du, Chuifeng Fan

**Affiliations:** Department of Pathology, First Affiliated Hospital and College of Basic Medical Sciences of China Medical University, 110001, Shenyang, China.

**Keywords:** β-catenin, NSCLC, progression, Ube2S, Wnt

## Abstract

Ubiquitin conjugating enzyme E2S (Ube2S) plays important roles in cancer development in some malignant tumors. However, the functions and related molecular network of Ube2S in non-small cell lung cancer are not fully understood. In the current study, we examined the expression of Ube2S in non-small cell lung cancer and its clinicopathological significance. We also investigated the molecules and pathways regulated by Ube2S. An immunostaining study showed that the positive rate of Ube2s expression in lung cancer tissues was higher than that in normal lung tissues (*p* < 0.05). Upregulated Ube2S expression in cancer tissues significantly correlated with clinical progression (TNM III versus I + II), lymph node metastasis, and shorter survival time of the patients (*p* < 0.05). When Ube2S was overexpressed in A549 cells, the abilities of these cells to proliferate and migrate were increased (*p* < 0.05). Moreover, Ube2S significantly upregulated the expression of β-catenin, cyclin D1, and MMP7 (novel molecules of the Wnt/β-catenin pathway), and the activity of this pathway (*p* < 0.05). In addition, a Wnt/β-catenin signaling inhibitor effectively abolished the function of Ube2S. These results indicate that Ube2S may be a novel marker contributing to lung cancer development, possibly through regulating canonical Wnt signaling.

## Introduction

Ubiquitination is an important process involved in a variety of cell functions, including cell signaling, mitosis, and endocytosis 1-3. It also plays roles in cancer development 4-8. Ubiquitin-conjugating enzyme E2S (Ube2S) belongs to the E2 family of proteins. It was initially found to be related to functions of the anaphase-promoting complex to control substrate ubiquitination 9,10. Ube2S was also found to be overexpressed in some cancers, including breast, hepatocellular, and endometrial cancer, and to be related to cancer progression 4-8. However, the function, and molecules and pathways regulated by Ube2S in non-small cell lung cancer (NSCLC), are not fully understood. In this study, we examined the expression of Ube2S in lung cancer and normal lung tissues, and investigated its impact on cancer cell biology, and related molecules and pathways.

## Materials and Methods

### Patients and tissue samples

Specimens included tumor and paired normal lung tissues. Patients did not receive any preoperative radiotherapy or chemotherapy. Pathological diagnoses were according to the World Health Organization (WHO) classification of tumors of the lung, pleura, thymus, and heart 11. Lung tumor histopathological types included squamous cell carcinomas (SCCs) and adenocarcinomas. We received approval from the Institutional Review Board of China Medical University for this study. Informed consent was obtained from all patients.

### Immunohistochemistry

The immunostaining assays were carried out and evaluated as described previously 12. An SP-kit was used according to the manufacturer's instructions. Tissue sections were incubated with a primary antibody against Ube2S (Santa Cruz, CA, USA, dilution, 1:100).

### Western blotting

The western blotting assays were carried out and evaluated as described previously 12. For western blotting, the antibodies and dilution factors used were as follows: Ube2S (1:500), β-catenin (1:500), cyclin D1 (1:500), MMP7 (1:200), myc-tag (1:500), and GADPH (1:500). All antibodies were from Santa Cruz Biotechnology (Santa Cruz, CA, USA).

### Cell culture and transfection

The cell lines studied included HBE, NCI-H1299, NCI-H719, and A549. Cells were cultured according to instructions of the ATCC/CTCC. Ube2S cDNA clone was purchased from Origene (Rockville, MD, USA). The Ube2s cDNA clone was transfected using Lipofectamine 2000 (Invitrogen, Carlsbad, CA, USA) according to the manufacturer's instructions.

### MTT assay

The MTT assay was described previously 13. Briefly, cell cultures were incubated with 3-(4,5-dimethylthiazol-2-yl)-2,5-diphenyl tetrazolium bromide (MTT) and the results were quantitated spectrophotometrically.

### Scratch wounding assay

The procedure of the assays has been described previously 12.

### Dual-luciferase assays

The assays were described previously 14.

### Statistical analysis

SPSS v13.0 (IBM, Chicago, IL, USA) was used for the statistical analyses. The clinicopathological significance of protein expression in tissues was determined using Pearson's chi-square test. Differences between the groups were determined using Student's t-test. *P*-values < 0.05 were considered significant.

## Results

### Ube2S expression and its clinicopathological significance in NSCLC

**Figure 1:**
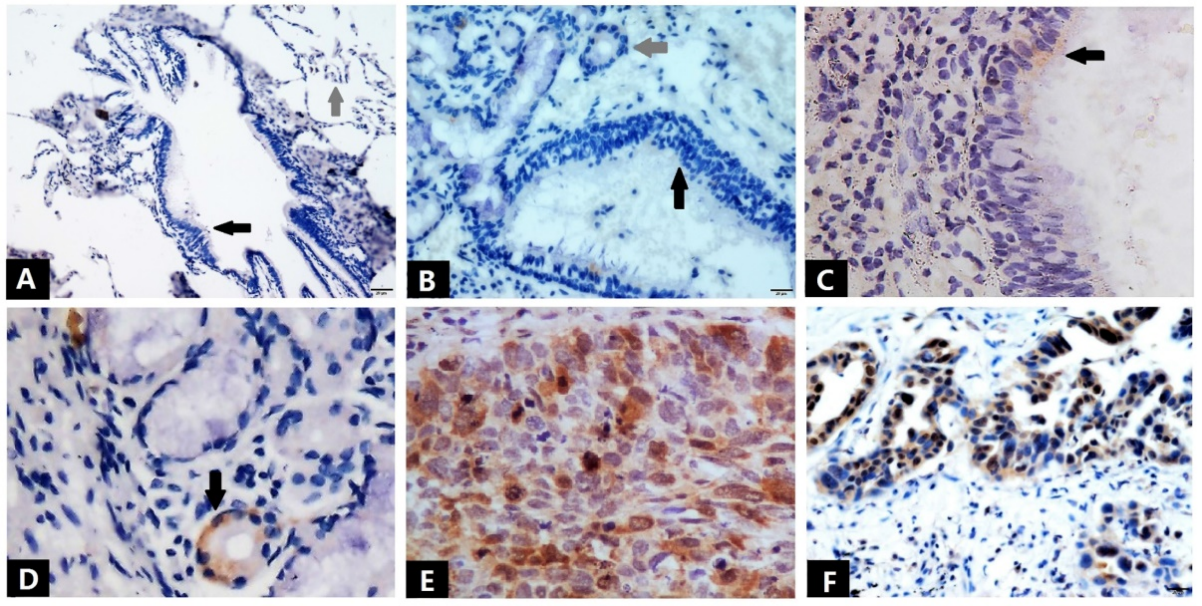
** Immunostaining of Ube2S in normal lung tissues and NSCLC tissues.** Bronchial epithelial cells (black arrow) and alveolar cells (grey arrow) show negative immunostaining for Ube2S (A). Bronchial epithelial cells (black arrow) and submucosal glands (grey arrow) show negative immunostaining for Ube2S (B). Weak and focal immunostaining of Ube2S is evident in some bronchial epithelial cells (C) and submucosal glands (D). Strong immunostaining of Ube2S is present in the cytoplasm and nuclei of squamous cell carcinoma (E) and adenocarcinoma (F) cells. (A: ×100; B, D, F: ×200; C, E: ×400)

**Figure 2:**
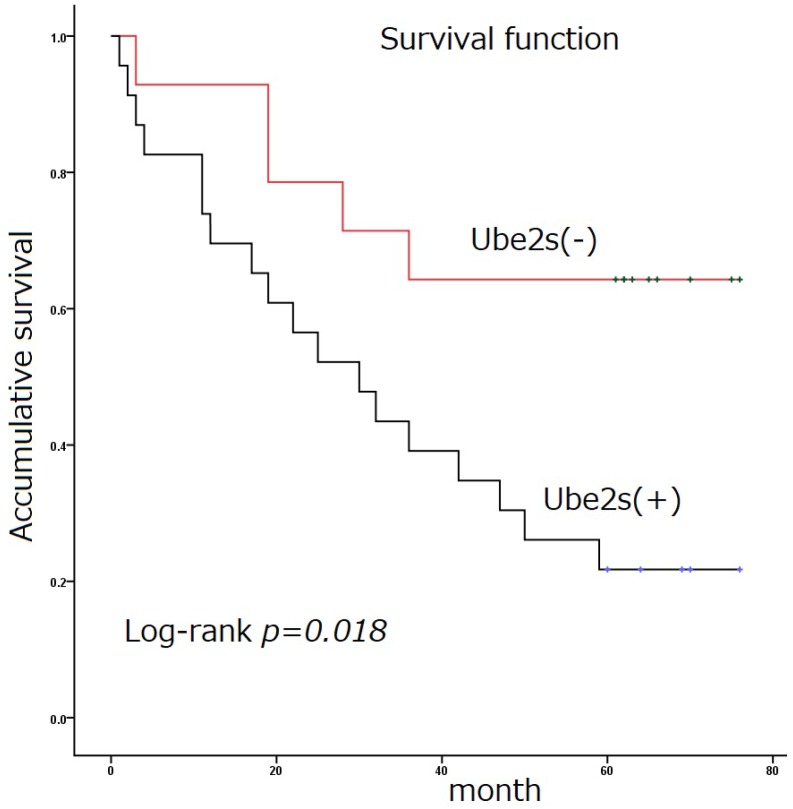
** Survival function.** Ube2S expression in non-small cell lung cancer is significantly associated with poor patient survival (34.9 ± 5.5 versus 56.4 ± 7.3 months) (Log rank test, *p* < 0.05)

Immunostaining was used to detect the expression of Ube2S in normal and tumor tissues (Figure 1 and Table 1). The immunostaining of Ube2S in normal lung tissues was weak, with a positive rate of 7.5% (3/40). The positive rate of Ube2S in cancer tissues was 59.8% (61/102), which was significantly higher than that in normal lung tissues (*p* < 0.05). Immunostaining of Ube2S was detected in the cytoplasm and nucleus. Elevated Ube2S expression significantly correlated with clinical progression of cancer (TNM III versus I + II). Ube2S expression was also detected more commonly in patients with lymph node metastases (*p* < 0.05). The mean survival time of patients expressing Ube2S in tumors was significantly shorter than that in patients without Ube2S expression (34.9 ± 5.5 versus 56.4 ± 7.3 months) (*p* < 0.05) (Figure 2).

### Ube2s promotes proliferation and migration of lung cancer cell in vitro

**Figure 3:**
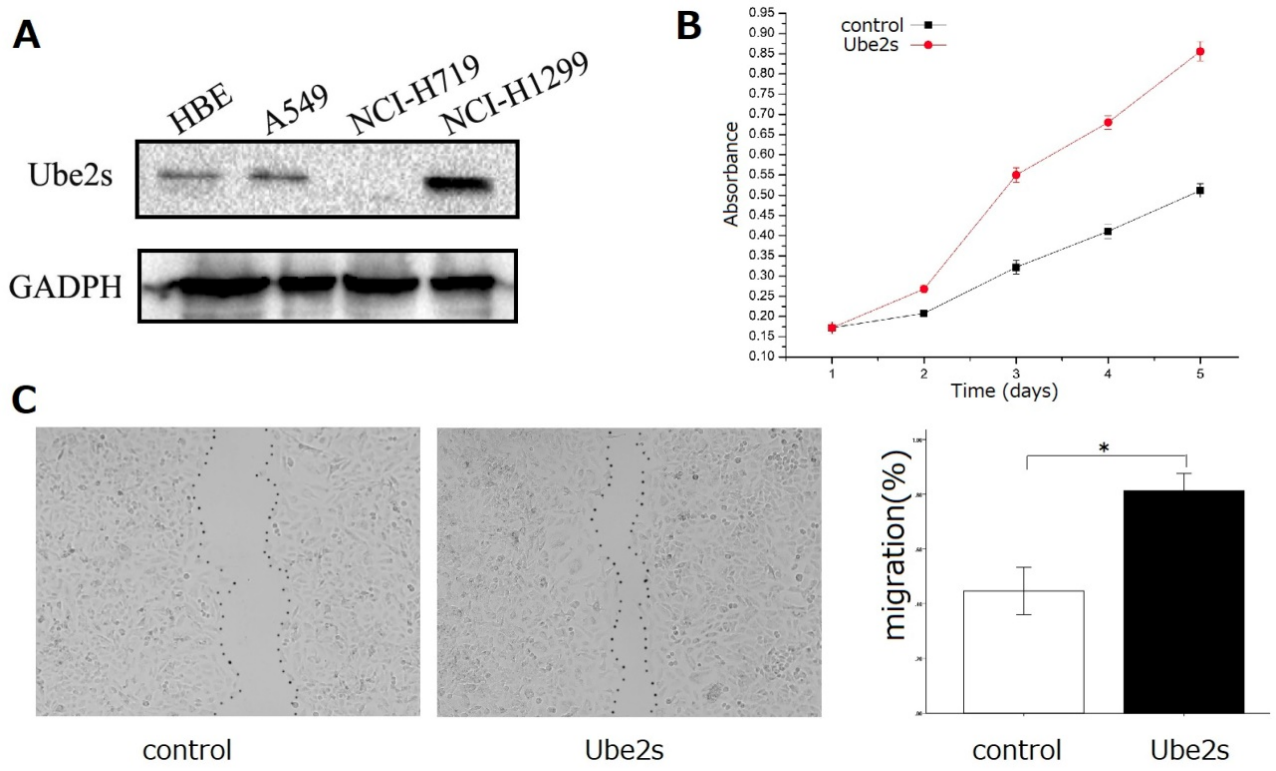
** The function of Ube2S in lung cancer cells.** Western blots show that Ube2S is expressed in bronchial epithelial HBE cells, and lung cancer A549 and NCI-H1299 cells (A). The MTT assay shows that overexpression of Ube2S in A549 cells significantly promotes cancer cell proliferation (B) (*p* < 0.05). The wound scratch healing assay shows that overexpression of Ube2S in A549 cells significantly promotes cancer cell migration (C) (*p < 0.05)

Western blotting showed that Ube2S was expressed in bronchial epithelial HBE cells and lung cancer cell lines including A549 and NCI-H1299 (Figure 3A). We selected A549 cells, which exhibited median Ube2S expression, for further investigation of the roles of Ube2S in cancer cells. We transfected Ube2S cDNA into A549 cells and found that overexpression of Ube2s significantly increased their proliferation (MTT assay, *p* < 0.05, Figure 3, B) and migration (wound scratch healing assay, *p* < 0.05, Figure 3C) abilities.

### Ube2S upregulates downstream molecules and the activity of Wnt/β-catenin signaling

**Figure 4:**
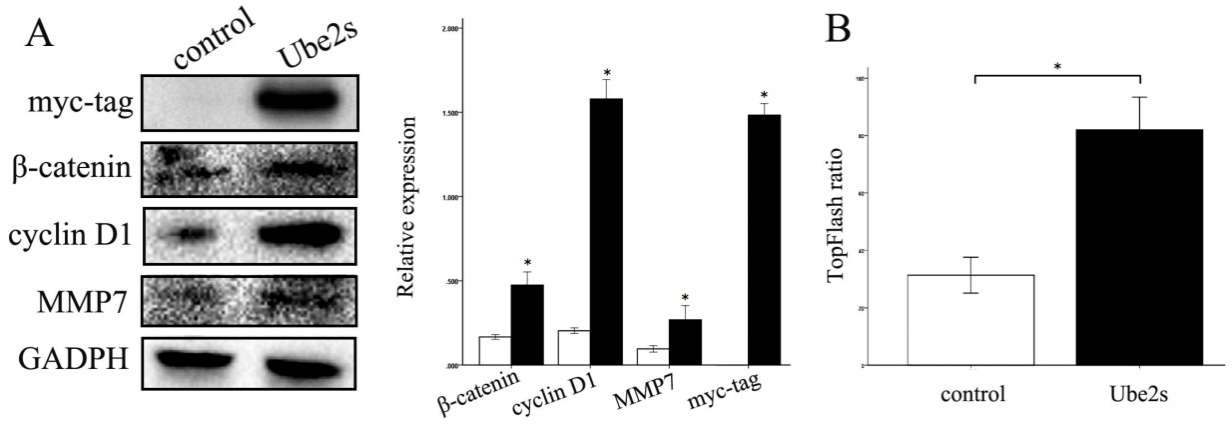
** Ube2S regulates Wnt/β-catenin signaling molecules and activity.** Western blots show that overexpression of Ube2S in A549 cells significantly upregulates Wnt/β-catenin signaling molecules including β-catenin, cyclin D1, and MMP7 (A) (*p* < 0.05). The luciferase assay shows that overexpression of Ube2S in A549 cells significantly upregulates Wnt/β-catenin signaling activity (B) (*p* < 0.05)

We next investigated the molecules and pathways involved in the regulation of cancer cell biology by Ube2S. Ube2S overexpression, by transfecting cDNA into cancer cells, significantly upregulated the expression of β-catenin, cyclin D1, and MMP7 proteins, novel members of the canonical Wnt signaling pathway (*p* < 0.05) (Figure 4A). The luciferase assay showed that Ube2S significantly upregulated the activity of the canonical Wnt pathway in cancer cells (*p* < 0.05) (Figure 4B).

### Wnt/β-catenin signaling inhibitor abolished the roles of Ube2S to regulate cancer cell biology

**Figure 5:**
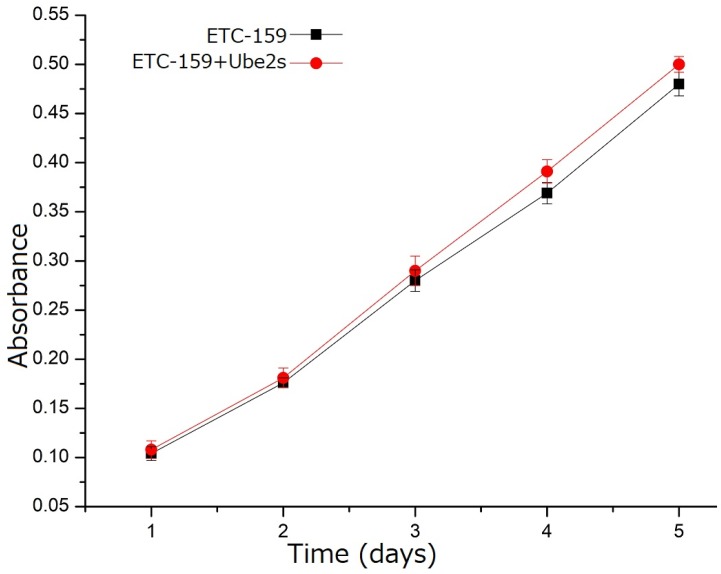
** Wnt/β-catenin signaling inhibitor abolished the function of Ube2s in lung cancer cells.** The MTT assay shows that addition of the Wnt/β-catenin signaling inhibitor, ETC-159, significantly inhibits the ability of Ube2S to promote cancer cell proliferation

We used the canonical Wnt pathway inhibitor, ETC-159, to inhibit the activity of this pathway in A549 cells to investigate whether the function of Ube2S in NSCLC depended on activating this pathway. The MTT assay showed that incubating A549 cells with ETC-159 significantly abolished the ability of Ube2S to promote cell proliferation (Figure 5).

## Discussion

Ubiquitination participates in regulating almost all life phenomena including the cell cycle, cell proliferation, differentiation, transcriptional regulation, and signal transduction, and also plays important roles in some pathological processes including carcinogenesis and cancer development 1-8. The ubiquitin-proteasome pathway is a common means of degrading endogenous proteins. This pathway involves the interaction of ubiquitin activator E1, ubiquitin binding enzyme E2, and ubiquitin ligase E3 1-4. Ube2S is an E2 enzyme that elongates ubiquitin chains on target proteins by interacting with the anaphase-promoting complex 9,10. However, the function of Ube2S in human malignancies, including NSCLC, is not fully understood.

Data regarding the expression of Ube2S in human tissues is limited. Some studies show that Ube2S is overexpressed in human malignancies and may contribute to cancer development. Ayesha et al. demonstrated that Ube2S was overexpressed in breast carcinoma and was associated with malignant characteristics 4. Yoshimura et al. found that Ube2S expression was elevated in oral squamous cell carcinoma and correlated with cancer cell proliferation 5. Pan's study indicated that Ube2S was highly expressed in hepatocellular carcinoma and correlated with cancer progression 7, while Lin et al. showed that Ube2S was overexpressed in endometrial cancer and contributed to the malignant phenotype 8.

In the current study, Ube2S was overexpressed in NSCLCs, including squamous cell carcinomas and adenocarcinomas, compared to normal lung tissues, including submucosal glands and bronchial epithelial tissues. The clinicopathological analysis showed that overexpression of Ube2S was significantly associated with lung cancer progression. This indicated that Ube2S may be a therapeutic target in this malignancy.

The molecular mechanism of Ube2S is not fully understood. In Yoshimura's study, P21 was regulated by Ube2S, which can explain its impact on cancer cell proliferation 5. Li et al. showed that β-catenin was modified by Ube2S, enhancing its stability and resulting in its accumulation in cancer cells to promote cancer development 6. Pan's study indicated that Ube2S contributed to cancer progression through promoting the ubiquitination and degradation of P53 7. SOX6 was also found to be regulated by Ube2S, which subsequently activated the canonical Wnt pathway 8.

In the current study, we investigated whether Ube2S regulated lung cancer cell proliferation through the canonical Wnt pathway. We found that Ube2S significantly upregulated the activity of the Wnt/β-catenin pathway and the expression of downstream molecules in A549 cells. ETC-159, an inhibitor of the Wnt/β-catenin pathway, significantly inhibited the ability of Ube2S to regulate cancer cell biology. This indicates that Ube2S may promote lung cancer progression by regulating canonical Wnt signaling. However, further investigations are needed to fully understand how Ube2S regulates β-catenin expression.

## Figures and Tables

**Table 1:** Clinical and pathological significance of Ube2s expression in NSCLC

Characteristics	Numbers	Negative Positive		p^*^
**Total**	102	41	61	
**Age (years)**				
<55	25	10	15	0.982
≥55	77	31	46	
**Gender**				
male	60	24	36	0.961
female	42	17	25	
**Histological type**				
squamous cell carcinoma	44	20	24	0.345
adenocarcinoma	58	21	37	
**Differentiation**				
well	20	11	9	0.132
Moderate and poor	82	30	52	
**TNM stage**				
Ⅰ and Ⅱ	61	34	27	0.000
Ⅲ	41	7	34	
**Lymph node metastasis**				
positive	45	10	35	0.001
negative	57	31	26	

* *p* values were obtained with the X^2^ test.

## Abbreviations

MTT: 3-(4,5-dimethylthiazol-2-yl)-2,5-diphenyl tetrazolium bromide
NSCLC: non-small cell lung cancer
SCCs: squamous cell carcinomas
Ube2S: Ubiquitin-conjugating enzyme E2S
WHO: World Health Organization
